# Quantitative assessment and determinants of the papillary microvasculature in healthy subjects

**DOI:** 10.1186/s12886-021-01896-5

**Published:** 2021-03-20

**Authors:** Li-jun Zhou, Xiu-zhi Luo, Pei-yang Shen, Xin Li, Peng Su, Zhe Zhu, Shi-gang Yan, Xiang-bin Kong, Xiao-he Lu

**Affiliations:** 1 Department of Ophthalmology, Zhujiang Hospital, Southern Medical University, Guangzhou, 515282 China; 2 Department of Ophthalmology, Affiliated Foshan Hospital, Southern Medical University, Foshan, 528000 China; 3 Department of Medicine, Division of Regenerative Medicine, University of California, San Diego, School of Medicine, La Jolla, CA 92037 USA

**Keywords:** Optical coherence tomography angiography, Superficial vessel density, Healthy subjects, Optic disc, Axial length

## Abstract

**Background:**

It is critical to monitor the optic disc’s vessel density using Optical coherence tomography angiography (OCTA) and evaluate its determinants. In the current study, we investigate the superficial vessel density (VD) of the papillary microvasculature and its determinants in healthy subjects of Southern China.

**Methods:**

This was a prospective, cross-sectional study. Superficial VD in healthy individuals’ optic disc region was measured by OCTA. The factors associated with ocular and systemic parameters were analyzed using a generalized estimation equation (GEE) model.

**Results:**

A total of 510 eyes of 260 healthy subjects were analyzed in the study. The total VD in the optic disc area was 17.21 ± 2.15 mm^− 1^ (95% CI, 17.02–17.40 mm^− 1^). The VD in the inner ring and the outer ring of the optic disc were significantly higher compared with the central ring, while the VD of the superior quadrant and inferior quadrant was significantly higher compared with the temporal and nasal quadrant. After adjusting for the ocular factors and systemic factors, AL (*β* = − 0.4917, *P* = 0.0003), disc area (*β* = − 0.3748, *P* = 0.0143), CMT (*β* = − 0.0183, *P* = 0.0003) and SSI (*β* = 1.0588, *P* < 0.001) were significantly associated with total VD of the optic disc.

**Conclusion:**

The mean total VD in the optic disc area was 17.21 ± 2.15 mm^− 1^ in healthy subjects, and the superior and inferior VD was significantly higher than the temporal and nasal VD. AL, disc area, CMT, and SSI may affect the total VD in the optic disc area and should be considered in clinical practice.

## Background

The optic disc, an important anatomic structure where the retinal nerve fiber converges, is a part of the nerve system. Blood flow plays an essential role in maintaining the normal function of the optic disc. Previous studies have demonstrated abnormal blood flow in optic neuropathies [1, 2]. Therefore, it is critical to quantify changes in microcirculation as an early marker for some diseases.

Fundus fluorescein angiography (FFA) has been the primary method used to examine the optic disc and retinal capillary circulation for many years [3]. However, FFA is an invasive examination and cannot quantify the vessel density (VD) [4]. Optical coherence tomography angiography (OCTA) is a recent non-invasive technology that can visualize and quantify the retinal and papillary microvasculature [5, 6]. Studies have demonstrated abnormal blood flow changes of the retina and optic discs in some diseases, including retinal vein occlusion [7], anterior ischemic optic neuropathy [8, 9], glaucoma [10], diabetic retinopathy [11, 12], and hypertension retinopathy [13].

However, there are scarce normative OCTA data from a healthy population, particularly concerning the optic disc’s VD [14, 15]. Additionally, there is little understanding of the determinants associated with papillary VD. Therefore, we performed this study to provide normative papillary VD data in healthy individuals and further investigated factors associated with ocular parameters and systematic parameters.

## Methods

### Ethical approval

Ethics approval was obtained from the Institutional Review Board of Foshan Second Hospital (KJ20190012). This was a cross-sectional study conducted at the Department of Ophthalmology, Foshan Second Hospital, Southern Medical University, between May 2019 and April 2020. This study met the Declaration of Helsinki’s tenets and was registered on the Chinese Clinical Trial Registry (identifier: ChiCTR1900024921). Informed consent was obtained from all subjects or, if subjects are under 18, from a parent or legal guardian before starting the study.

### Study population

Healthy subjects who had a best-corrected visual acuity (BCVA) of 20/25 or better without clinical evidence of ocular diseases were included in the study. The inclusion criteria were as follows: intraocular pressure (IOP) of less than 21 mmHg, normal findings of the slit lamp and fundus examination. The exclusion criteria were: a history of ocular surgery or ocular trauma, significant opacity media preventing high-quality imaging, glaucoma, systemic diseases, such as hypertension, diabetes mellitus, retinal and choroidal disease, for example, retinal vein obstruction, macular hole, and choroidal retinopathy.

### Ophthalmic examinations

All subjects underwent complete ophthalmic examinations, including BCVA, slit-lamp examination, spherical equivalent (SE), IOP, fundus evaluation, and OCTA examination. Ocular biometric parameters, including central corneal thickness (CCT), axial length (AL), anterior chamber depth (ACD), and lens thickness (LT), were performed using optical low-coherence reflectometry (LenStar LS 900, Haag-Streit, Inc., Koeniz, Switzerland). The central macular thickness (CMT, central 1 mm) was assessed using the macular cube 512 × 128 model of the optical coherence tomography (Cirrus 5000 HD-OCT; Carl Zeiss Meditec, Inc., Dublin, CA). Disc area and cup area were measured by a 200 X 200 optic disc cube scan. Arterial blood pressure, body weight, and height were recorded. The ocular perfusion pressure (OPP) was calculated according to the equations: OPP = 2/3(mean arterial pressure - IOP), where mean arterial pressure = 1/3systolic pressure + 2/3 diastolic blood pressure. The body mass index (BMI) was also calculated as a variable to explore its association with the optic disc VD.

**Fig. 1:**
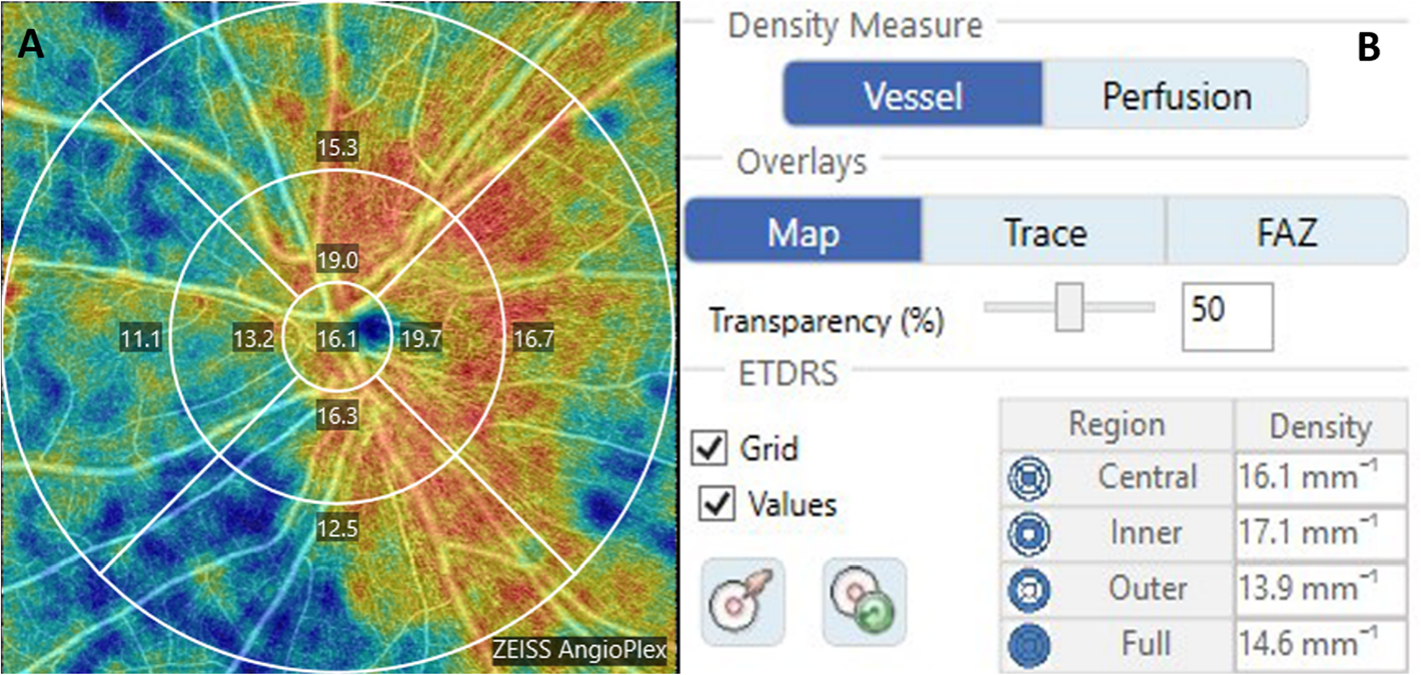
Vessel density of superficial capillary in the optic disc. **a** Shows four quadrants’ vessel density (superior, temporal, inferior, and nasal) in the inner ring and outer ring. **b** Shows the vessel density of different rings (central, inner, outer, and full)

OCTA examination was performed for all subjects using the Zeiss Cirrus HD-OCT 5000 and Angioplex device. To obtain the VD of the superficial capillary plexus (SCP) on the optic disc, the scanning model 6X6 mm was used with a real-time eye-tracking system. The SCP was measured from the internal limiting membrane (ILM) to the inner plexiform layer (IPL). The VD was defined as the total length of perfused vasculature per unit area (mm^− 1^). The papillary VD was automatically divided into four sections: the central ring, inner ring, outer ring, and full circle (Fig. 1a) and was calculated automatically by the OCTA software. Each ring was grouped into four quadrants based on the Early Treatment of Diabetic Retinopathy Study. In this study, the total VD referred to the VD in the full circle area (Fig. 1b). The images of OCTA with a quality signal strength of 7 or higher were analyzed.

### Statistical analysis

Data were presented as mean ± standard deviation (SD) for continuous variables and frequency (%) for categorical variables. For associated factors of total papillary VD, regression analysis was performed using a generalized estimation equation (GEE) model that was applied to adjust the possible intra-eyes correlation. Firstly, univariate GEE was performed to identify potential prognostic factors. The dependent variables, including systematic parameters and ocular parameters, were selected into the models, respectively. Then, variables with *P* < 0.30 in univariate analysis and potential confounders (age and gender) were included in the multivariate GEE model. Regression coefficients with 95% confidence intervals (CI) were presented. SAS statistical software version 9.4 (SAS Institute Inc., Cary, NC) was used for statistical analyses. A *P*-value < 0.05 was considered statistically significant.

## Results

**Fig. 2:**
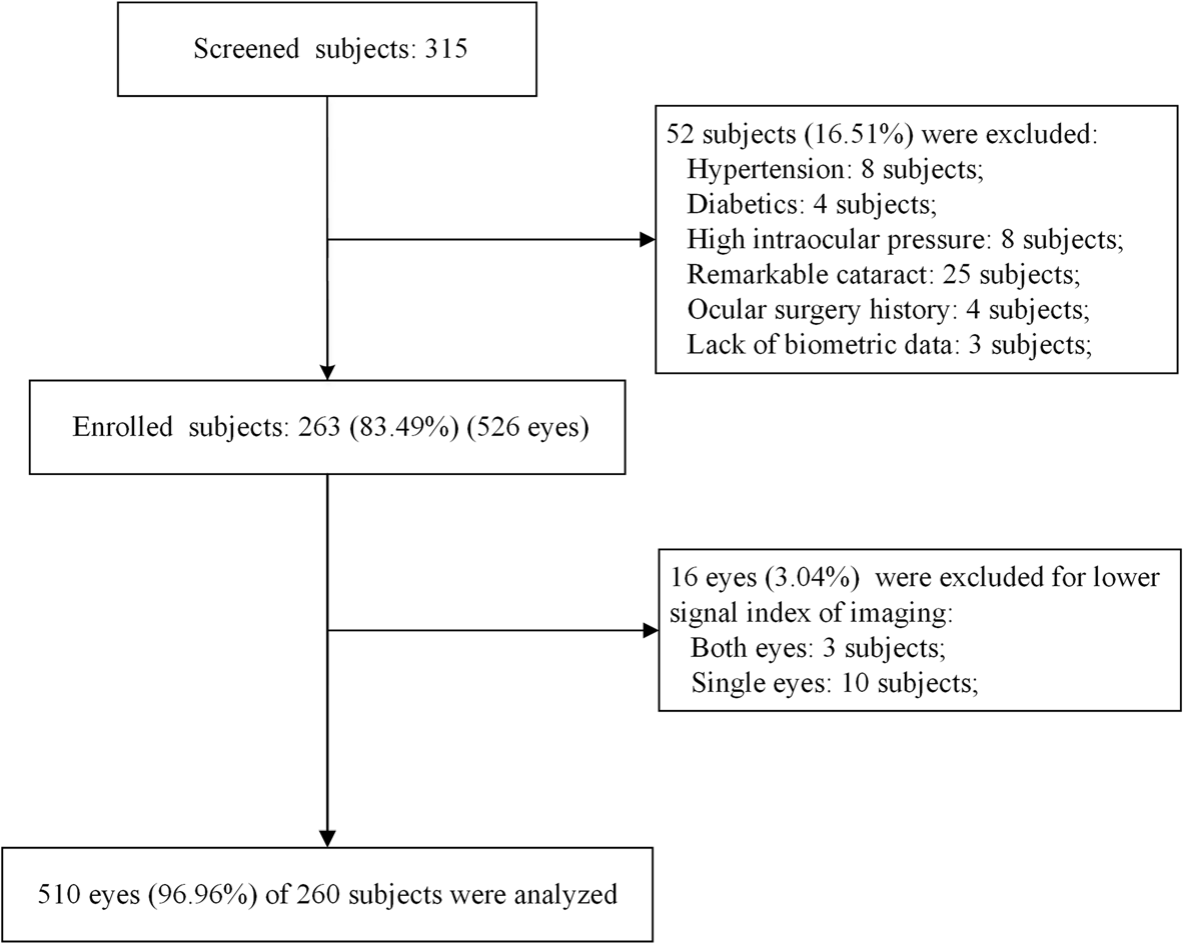
Flowchart of inclusion and exclusion

Of 315 screened individuals, a total of 263 subjects (83.49%) were enrolled in the study. Fifty-two subjects were excluded from analysis, including 8 subjects with hypertension, 4 subjects with diabetics, 8 subjects with high IOP, 25 subjects with remarkable cataract, 4 subjects with an ocular surgery history, 3 subjects with lack of refraction or biometric data. Additionally, of the enrolled 526 eyes of 263 individuals, 16 eyes were excluded because the imaging signal strength index was less than 7, including 3 bilateral individuals and 10 unilateral individuals. Finally, the 510 eyes of 260 individuals remaining subjects were analyzed in this study (Fig. 2).

The subjects’ mean age was 38.32 ± 21.89 years (95% CI, 35.64 – 40.99, range, 5-84 years), and 123 (47.31%) subjects were male. All demographic data were presented in Table 1.

**Table 1:** Demographic and clinical characteristics of study participants

Variables	Means ± SD	95% CI
Age (years)	38.32 ± 21.89	35.64–40.99
Body weight (kg)	52.59 ± 14.13	50.87–54.32
Body height (cm)	158.25 ± 11.62	156.83–159.67
BMI (kg/m^2^)	20.64 ± 3.89	20.16–21.11
Systolic (mmHg)	114.36 ± 16.01	112.40–116.31
Diastolic (mmHg)	72.39 ± 10.12	71.16–73.63
OPP (mmHg)	42.87 ± 8.09	41.88–43.86
SE (diopter)	−1.62 ± 2.52	−1.84 - -1.40
IOP (mmHg)	14.82 ± 2.72	14.59–15.06
CCT (μm)	537.86 ± 31.83	535.09–540.63
ACD (mm)	3.40 ± 0.43	3.36–3.44
LT (mm)	4.01 ± 0.57	3.96–4.06
AL (mm)	24.10 ± 1.21	24.00–24.21
CMT (μm)	279.99 ± 13.91	278.78–281.20
SSI	9.95 ± 1.10	8.86–9.05
Disc area (mm^2^)	1.91 ± 0.46	1.87–1.95
Cup area (mm^2^)	0.50 ± 0.40	0.46–0.53

*BMI* body max index, *OPP* ocular perfusion pressure, *SE* spherical equivalent, *IOP* intraocular pressure, *CCT* central corneal thickness, *ACD* anterior chamber depth, *LT* lens thickness, *AL* axial length, *CMT* central macular thickness, *SSI* signal strength index

**Fig. 3:**
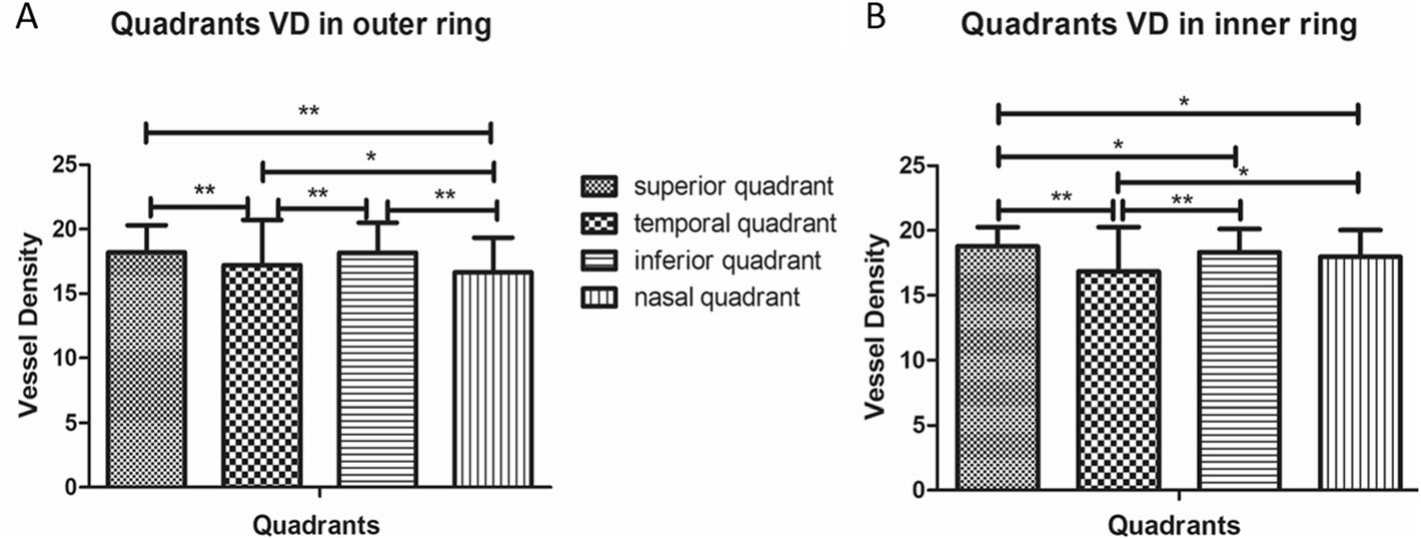
Comparison of vessel density of different quadrants in the outer (**a**) and the inner ring (**b**)

The mean VD was 5.28 ± 4.30 mm^− 1^ (95% CI, 4.91–5.66 mm^− 1^) in the center ring, 18.15 ± 3.34 mm^− 1^ (95% CI, 17.86–18.44 mm^− 1^) in the inner, and 17.53 ± 2.93 mm^− 1^ (95% CI, 7.27–17.78 mm^− 1^) in the outer, respectively. The VD of the inner and outer was significantly higher compared with the center (*P* < 0.001), but no significant difference was found between the inner ring and outer ring (*P* = 0.8992). In the outer ring (Fig. 3a) and the inner ring (Fig. 3b) of the optic disc OCTA, the VD in the superior quadrant and inferior quadrant was statistically higher than the temporal and nasal quadrant.

The mean superficial VD in the optic disc’s full circle was 17.20 ± 2.15 mm^− 1^(95% CI, 17.02–17.40 mm^− 1^; range, 5.30–19.70 mm^− 1^). Univariate regression analysis showed that the SE (*β* = 0.2018, *P* < 0.001), the AL (*β* = − 0.5477, *P* < 0.001), the CMT (*β* = 0.0232, *P* = 0.0006), and the SSI (*β* = 1.0832, *P* < 0.001) were associated factors, which was significantly affected the papillary VD of SCP in healthy individuals. Besides, the papillary VD was not significantly associated with age (*P* = 0.7771), gender (male vs. female, *P* = -0.2956), body weight (*P* = 0.6156), body height (*P* = 0.0554), BMI (*P* = 0.6145), systolic pressure (*P* = 0.5152), diastolic pressure (*P* = 0.5182), IOP (*P* = 0.2579), CCT (*P* = 0.8471), ACD (*P* = 0.1074), LT (*P* = 0.2778), disc area (*P* = 0.2510) and cup area (*P* = 0.9771) (Table 2).

**Table 2:** Univariate analysis of the associated factors of total vessel VD in the optic disc

Variables	β (95% CI)	*P*-value
Age (year)	0.0005 (− 0.0008–0.0117)	0.7771
Gender (male vs. female)	−0.2358 (− 0.6777–0.2061)	0.2956
Body weight (kg)	−0.0041 (− 0.0200–0.0119)	0.6156
Body height (cm)	−0.0193 (− 0.0390–0.0004)	0.0554
BMI (kg/m^2^)	0.0145 (− 0.0420–0.0711)	0.6145
Systolic pressure (mmHg)	−0.0045 (− 0.0181–0.0091)	0.5152
Diastolic pressure (mmHg)	0.0069 (− 0.0141–0.0279)	0.5181
OPP (mmHg)	0.0066 (− 0.0185–0.0318)	0.6045
SE (diopter)	0.2018 (0.1015–0.3021)	< 0.001
IOP (mmHg)	− 0.0462 (− 0.1261–0.0338)	0.2579
CCT (μm)	−0.0007 (− 0.0074–0.0061)	0.8471
ACD (mm)	−0.3663 (− 0.8121–0.0796)	0.1074
LT (mm)	0.2024 (− 0.1631–0.5680)	0.2778
AL (mm)	−0.5477 (− 0.7533- -0.3422)	< 0.001
CMT (μm)	0.0232 (0.0100–0.0365)	0.0006
SSI	1.0832 (0.9082–1.2582)	< 0.001
Disc area (mm^2^)	0.2475 (−0.1751–0.6702)	0.2510
Cup area (mm^2^)	0.0076 (− 0.5128–0.5281)	0.9771

*CI* confidence intervals, *BMI* body max index, *OPP* ocular perfusion pressure, *SE* spherical equivalent, *IOP* intraocular pressure, *CCT* central corneal thickness, *ACD* anterior chamber depth, *LT* lens thickness, *AL* axial length, *CMT* central Macular thickness, *SSI* signal strength index

Variables with P < 0.30 in univariable analysis and potential confounders (age, gender) were included in multivariate analysis models. AL (*β* = − 0.4917, *P* = 0.0003) and disc area (*β* = − 0.3748, *P* = 0.0143) were significantly negatively associated with total VD of the optic disc, while CMT (*β* = − 0.0183, *P* = 0.0003) and SSI (*β* = 1.0588, *P* < 0.001) were positively related to total VD of the optic disc (Table 3). However, after controlling the ocular parameters and systemic factors, age, gender, body height, SE, ACD, and LT were not significantly associated with the optic disc’s total VD (Table 3).

**Table 3:** Multivariate analysis of the associated factors of total vessel VD in the optic disc

Variable	β (95% CI)	*P*-value
Age (year)	0.0057 (− 0.0130–0.0243)	0.5523
Gender (male vs. female)	− 0.2265 (− 0.6005–0.1475)	0.2353
Body height (cm)	−0.0158 (− 0.0323–0.0006)	0.0588
SE (diopter)	−0.0096 (− 0.0096–0.1089)	0.8743
IOP (mmHg)	−0.0224 (− 0.0866–0.0417)	0.4929
ACD (mm)	0.4096 (− 0.1858–1.0050)	0.1775
LT (mm)	−0.3154 (− 0.9939–0.3630)	0.3622
AL (mm)	−0.4917 (− 0.7552 - -0.2281)	0.0003
CMT (μm)	0.0183 (0.0083–0.0282)	0.0003
SSI	1.0588 (0.8983–1.2193)	< 0.001
Disc area (mm^2^)	−0.3748 (− 0.6745 - -0.0750)	0.0143

*CI* confidence intervals, *SE* spherical equivalent, *IOP* intraocular pressure, *ACD* anterior chamber depth, *LT* lens thickness, *AL* axial length, *CMT* central Macular thickness, *SSI* signal strength index

## Discussion

Normative data of papillary VD is needed and has important implications in understanding the development of optic neuropathies, for example, optic neuritis, anterior ischemic optic neuropathy, glaucoma, and other diseases [9, 16–18]. This study investigated the papillary VD of SCP and its determinants in healthy individuals aged 5 to 84. The results showed that the mean total VD of the papillary area was 17.21 ± 2.15 mm^− 1^ (95% CI, 17.02–17.40 mm^− 1^). AL, CMT, SSI, and disc area significantly affected the total VD after adjusting for the confounders. Additionally, the center ring’s VD was considerably lower than that in the inner and outer regions. The VD of the superior sector and inferior sectors was higher compared with the temporal and nasal sector.

There were different VD data of superficial capillary (SCP) in the optic disc from healthy individuals using other OCTA devices. Fernandez-Vigo et al. measured the optic nerve head VD using swept-source optical coherence tomography angiography. They reported that the VD for central SCP was 45.3%. The VD in vertical quadrants (60.5 and 61.9% in superior and inferior, respectively) was higher than the horizontal quadrants (53.4 and 52.4% in temporal and nasal, respectively) [19]. However, Zhang et al. found no difference between superior and inferior peripapillary areas in 71 healthy subjects aged 5 to 18 [20]. Bazvand et al. used the RTVue XR Avanti (Optovue, Fremont, CA, USA) to measure the peripapillary VD in 79 healthy eyes of individuals with a mean age 37.03 ± 11.27 years (12 to 67 years). They found that the total VD of the optic disc was 56.03% ± 4.55% [17]. A study with a large sample size of 346 eyes also revealed that the peripapillary VD was 57.2 ± 5.7% for SCP, measured by the swept-source optical coherence tomography angiography (SS-OCTA). Further analysis showed that vertical peripapillary VD was significantly higher compared with the horizontal measurements [15]. In our study, a similar finding was observed that VD in the superior quadrant and inferior quadrant was significantly higher than the temporal and nasal quadrants. Why did the VD vary from studies? A possible explanation was that there could have been a lack of standardization of data due to different OCTA devices in those studies. Besides, there was a difference in the range and depth of the optic disc scanned by OCTA. Enrolled subjects also differed in each study. Additionally, each individual varies in the disc-fovea axis, impacting the asymmetry of retinal fiber layer (RNFL) thickness [21]. Greater RNFL thickness should require more blood flow to meet the need for tissue metabolism. As a result, the vessel density is positively related to the RNFL thickness [22].

Different determinants affecting papillary VD have been reported in recent studies. The impact of age and gender on the VD of the optic disc remains controversial. Several studies demonstrated a significant impact of age and gender on the VD, while others reported no influence. Wang et al. and Jo et al. observed that the VD decreased with age [23, 24]. Similarly, Zhang et al. also reported that age could account for 15% of VD variations [20]. However, Rao et al. found that age did not influence the papillary VD [14]. Chang et al. found that male sex was associated with reduced VD [25]. In contrast, Mansoori et al. found no association between sex and papillary VD in 52 healthy subjects using RTVue XR 100 Avanti OCT [26]. Another study also showed no association between sex and VD of the papillary area [15]. Consistent with those results, age and sex were not significantly associated with the optic disc’s superficial VD in the present study. One possible explanation for these different results is that those studies varied from inclusion criteria of subjects, especially in age. Besides, various machines and scan models also could account for the discrepancy to some extent.

Several authors have investigated the association between ocular parameters and VD. Nelson et al. found that AL was negatively associated with peripapillary VD in 1029 eyes from 1029 subjects [27]. Other studies also demonstrated similar results [28, 29]. A decrease in total papillary VD with AL (β = − 0.4917, *P* = 0.0003) was found in this study after controlling other variables. A significantly positive association between the mean macular thickness and total papillary VD was found in this study. A possible explanation may be that a thicker retina has a higher demand for nutrients and oxygen [30]. Besides, the multivariate analysis demonstrated that disc area was negatively associated with total VD, which is consistent with previous findings [31].

Systemic factors, such as blood pressure, are expected to affect VD [14, 32]. Interestingly, blood pressure, including diastolic pressure, systolic pressure, and OPP, was not significantly associated with papillary VD in healthy subjects in the present study. Similar findings are demonstrated by Rao et al. and Liu et al. [16, 25]. We speculate that it may be related to the autoregulation of blood pressure. Given the healthy individuals in our study, blood pressure and OPP changes did not significantly affect the ocular blood.

It is worth noting that SSI is significantly associated with the VD in optic disc both in the univariate model and multivariate. To ensure imaging quality and reduce the impact of SSI on VD, we only enrolled the eyes with SSI greater than 7. Even so, SSI still has a more substantial effect on the VD. The result was consistent with previous studies [33, 34]. Therefore, the SSI should be taken into consideration when measuring the VD by OCTA.

This study also has limitations. Firstly, we have not performed a correction of our data for potential magnification in the image sizes. Axial length may lead to changes in image size magnification, especially in myopia. The true size of the fundus area imaged is related to the magnification of the camera and eye [35]. Axial length variation also has an impact on the superficial VD [36]. There are some individuals with myopia in our study. In the follow-up work, we need to investigate its effects on VD further. Secondly, the disc’s large retinal vessel is not removed automatically in our study, which can generate projection artifacts on the deeper layer and impact on the VD. However, we focus on the superficial VD in healthy individuals. So it maybe has little effect on superficial VD in our study [37, 38]. Additionally, subjects younger than 5 years are not included in the present study because they are likely not to finish the OCTA examination for high-quality imaging. Finally, although the study presents a normative database from Southern China, it may not represent the entire population.

In conclusion, using a relatively large sample size (510 eyes), the superficial VD measurement of the optic disc is measured using OCTA. The correlation from both eyes of a person is adjusted with the GEE regression model. We found that the mean of the total VD in the optic disc area was 17.21 ± 2.15 mm^− 1^ (95% CI, 17.02–17.40 mm^− 1^). The univariate analysis shows that the body height, the SE, the AL, and the CMT are associated with total VD. However, after adjusting for other variables, the multivariate regression model showed that the AL, CMT, SSI, and disc area are significantly related to total papillary VD. Therefore, when evaluating the optic disc’s superficial VD in clinical practice, these parameters should be taken into account.

## Data Availability

The datasets used in the current study are available from the corresponding author on a reasonable request.

## Abbreviations

ACD: Anterior chamber depth
AL: Axial length
BCVA: Best-corrected visual acuity
BMI: Body mass index
CCT: Central corneal thickness
CI: Confidence intervals
CMT: Central macular thickness
FFA: Fundus fluorescein angiography
GEE: Generalized estimation equation
IOP: Intraocular pressure
OCTA: Optical coherence tomography angiography
OPP: Ocular perfusion pressure
SCP: Superficial capillary plexus
SD: Standard deviation
SE: Spherical equivalent
SSI: Signal strength index
VD: Vessel density
